# Curcumin Synergistically Sensitizes Multidrug-Resistant Lung Cancer to Doxorubicin Through Ferroptosis-Associated Oxidative Stress

**DOI:** 10.3390/antiox15030288

**Published:** 2026-02-26

**Authors:** Wing-Hin Lee, Ching-Yee Loo, Poh Yen Khor, Charles Gnanaraj, Cai Ping Koh, Chean Ring Leong, Kamal Dua, Stewart Yeung, Kit-Leong Cheong

**Affiliations:** 1 College of Food Science and Technology, Guangdong Ocean University, Zhanjiang 524088, China; cyloo@unikl.edu.my; 2 Faculty of Pharmacy and Health Sciences, Royal College of Medicine Perak, Universiti Kuala Lumpur (UniKL RCMP), Ipoh 30450, Perak, Malaysia; pykhor@unikl.edu.my (P.Y.K.); charles.gnanaraj@unikl.edu.my (C.G.); 3 Faculty of Medicine, Quest International University, Ipoh 30250, Perak, Malaysia; pinko.koh@qiu.edu.my; 4 Centre for Stem Cell Research, Quest International University, Ipoh 30250, Perak, Malaysia; 5 Malaysian Institute of Chemical and Bioengineering Technology, Universiti Kuala Lumpur, Alor Gajah 78000, Melaka, Malaysia; crleong@unikl.edu.my; 6 Discipline of Pharmacy, Graduate School of Health, University of Technology Sydney, Ultimo, NSW 2007, Australia; kamal.dua@uts.edu.au (K.D.); stewart.yeung@uts.edu.au (S.Y.); 7 NICM Health Research Institute and School of Science, Western Sydney University, Westmead, NSW 2145, Australia; 8 Woolcock Institute of Medical Research, Macquarie University, Sydney, NSW 2113, Australia; 9 Australian Research Centre in Complementary and Integrative Medicine, Faculty of Health, University of Technology Sydney, NSW 2007, Australia

**Keywords:** oxidative stress, ferroptosis, curcumin, doxorubicin, lung cancer, multidrug resistance

## Abstract

Excessive oxidative stress can cause irreversible cytotoxic damage to both healthy and cancer cells through the induction of reactive oxygen species (ROS) mediated lipid peroxidation. Ferroptosis has recently been shown to promote lipid peroxidation due to the over-accumulation of iron. Although cancer cells possess elevated antioxidant capacity to neutralize chemotherapy-induced oxidative stress, the co-delivery of polyphenol compounds such as curcumin (CUR) can overwhelm these defenses by elevating intracellular ROS levels to a toxic threshold, thereby increasing anticancer efficacy. In this study, we evaluated the potential of CUR to chemosensitize doxorubicin (DOX) towards the DOX-resistant lung cell line (H69AR). Our results demonstrated that the combination of DOX and CUR resulted in a concentration-dependent behavior, where low-dose concentrations exhibited antagonistic effects, while high-dose IC_50_-equivalent concentrations shifted towards synergism. The combination induced significantly greater mitochondrial dysfunction, ATP depletion, cytochrome C release, and caspase-3 activation. This also resulted in excessive ROS generation, intracellular iron overload, and lipid peroxidation, accompanied by a reduction in antioxidant enzymatic activities. Pretreatment with N-acetyl-L-cysteine (ROS inhibitor) and ferrostatin-1 (ferroptosis inhibitor) further supported the involvement of oxidative stress and ferroptosis in modulating apoptosis and DNA fragmentation. Molecular docking analyses supported the binding of CUR and DOX to key ferroptosis regulators. This study shows the potential of CUR to sensitize DOX-resistant cancer cells through ferroptosis-linked-oxidative stress targeting.

## 1. Introduction

Despite extensive efforts to understand the molecular mechanisms underlying lung cancer, this remains the leading cause of cancer-related mortality worldwide. Chemotherapy remains one of the essential treatments for lung cancer. To date, many chemotherapeutic agents are clinically used, which include platinum-based drugs, taxanes, antimetabolites, topoisomerase inhibitors, and vinca alkaloids [1]. In addition, combination therapies that integrate chemotherapies and immunotherapies (e.g., atezolizumab, pembrolizumab, and durvalumab) or targeted therapies (e.g., osimertinib and erlotinib) have further enhanced therapeutic outcomes by improving tumor targeting and reducing systemic toxicity.

The incidence of multi-drug resistance (MDR) is implicated in over 90% of treatment failures [2]. Although different treatment modalities are employed to overcome MDR in cancers, these approaches often induce cross-resistance to structurally and mechanistically distinct chemotherapeutic agents, thereby further reducing the available treatment options [3]. P-glycoprotein (P-gp) and multidrug resistance-associated proteins are the most extensively characterized MDR proteins [4,5]. Both proteins actively pump out chemotherapeutic agents from cancer cells, therefore confer resistance to structurally diverse drugs. Additionally, cancer cells acquire the ability to repair DNA damage, inhibit senescence, and inhibit the apoptotic pathway to enable sustained uncontrolled proliferation [6].

Although doxorubicin (DOX) is associated with severe toxicity that can lead to heart failure, it remains a widely used chemotherapeutic agent to treat lung, breast, ovarian, gastric, and thyroid cancers [7]. The anticancer mechanisms of DOX include DNA intercalation, inhibition of DNA/RNA synthesis, suppression of topoisomerase II, reactive oxygen species (ROS) production, and promotion of cell cycle arrest [7]. However, cancer cells develop resistance to DOX rapidly, which is governed by overexpression of P-gp and BCL-2, reduced tumor suppressor protein (TP53) activity, and altered topoisomerase II function, which significantly compromises therapeutic effectiveness [8]. Therefore, this signals an urgent need for alternative treatment strategies to overcome DOX resistance.

Curcumin (CUR), a polyphenol derived from turmeric, a common dietary compound that exhibits wide therapeutic uses, including cancer treatment, owing to its potent antioxidant and anti-inflammatory effects [9]. The ability of CUR to selectively target cancer cells has been demonstrated across multiple cancer types [10,11,12,13]. Clinical investigations of CUR in patients with pancreatic cancer have shown promising results, with a reduction in cyclooxygenase-2 (COX-2) and NF-κB levels [14]. In addition, a higher objective response rate (ORR) was observed in breast cancer patients [15]. In lung cancer, CUR is effective in suppressing metastasis and MDR via the modulation of E-cadherin, matrix metalloproteinase, signal transducer and activator of transcription 3 (STAT3), PI3K/Akt/mTOR, and epidermal growth factor receptor (EGFR) [16].

Recent studies highlighted CUR as a ferroptosis inducer, promoting programmed cancer-cell death, mainly through iron-dependent lipid peroxidation. Antioxidant enzymes such as superoxide dismutase (SOD), glutathione (GSH), and glutathione peroxidase 4 (GPX4) are insufficient to neutralize the excessive iron-mediated oxidative stress [17]. Free iron (Fe^2+^) reacts with lipid hydroperoxides to produce highly reactive alkoxyl radicals that attack polyunsaturated fatty acids (PUFAs) in the cell membrane, leading to lipid peroxidation, membrane destabilization, and ultimately cell death [17]. Recent studies have shown that CUR can inhibit cell proliferation by activating the ferroptosis pathway [18]. Therefore, CUR shows high potential to target the ferroptosis-MDR axis owing to its ability to suppress P-gp expression and inhibit NF-κB, p-STAT3, and PI3K/Akt/mTOR signaling pathways, as well as disrupting iron metabolism and impairing the GSH-GPX4 antioxidant system [19].

However, the mechanistic study evaluating the crosstalk between ferroptosis and MDR signaling in lung cancer following CUR treatment remains limited. We hypothesize that CUR overcomes DOX resistance in lung cancer cells by exploiting ferroptosis, leading to suppression of proliferation in DOX-resistant H69AR cells through disruption of antioxidant defences and redox homeostasis. Therefore, in this study, we aim to evaluate the potential of CUR in regulating the proliferation of DOX-resistant lung cancer cell lines (H69AR), focusing on the mechanisms and their relationship with antioxidant systems.

## 2. Materials and Methods

### 2.1. Materials

CUR, doxorubicin hydrochloride (DOX), ferrostatin-1 (FER-1), N-acetyl-L-cysteine (NAC), dimethylsulfoxide (DMSO), 2′,7′-dichlorofluorescein diacetate (2′,7′-DCFA), lipopolysaccharide (LPS), bicinchoninic acid kit (BCA), CelLytic^TM^ M cell lysis reagent, protease inhibitor, mitochondrial membrane potential kit (MAK159), and iron assay kit (MAK472) were purchased from Sigma-Aldrich, Petaling Jaya, Malaysia. RPMI-1640 medium, trypsin-EDTA solution, fetal bovine serum (FBS), Annexin V-APC, and 7-aminoactinomycin D (7AAD) were purchased from Thermo Fisher Scientific, Shah Alam, Malaysia. CellTiter 96 MTS reagent was purchased from Promega Corporation, Madison, WI, USA. All cell culture plasticware was supplied by Matrioux, Kuala Lumpur, Malaysia. Analytical grade acetonitrile and absolute ethanol were used without further purification.

### 2.2. Molecular Docking Analysis to Determine the Ferroptosis Properties of CUR and DOX

The 3D structures of CUR and DOX were drawn and built using ChemDraw and ChemDraw3D Professional version 25. The structure was geometrically optimised by energy minimisation using the MM2 Force Field. Using AutoDockTools 1.5.6 “http://mgltools.scripps.edu/down-loads/ (accessed on 11 November 2025)”, the partial atomic charges were added to the ligand, and the Gasteiger atom charges were added to the protein macromolecule. Protein target as receptors for Acsl4 (Acyl-CoA synthetase long-chain family member 4, PDB ID:8w0u); GPX4 (Glutathione peroxidase 4, PDB ID: 6hkq); Nrf2 (Nuclear factor erythroid 2- related factor 2, PDB ID:2dyh); SLC7A11 (Solute carrier family 7 member 11, UniProt ID: Q9WTR6) and TFR1 (Transferrin receptor, PDB ID: 1suv) were obtained from the UniProt “https://www.uniprot.org (accessed on 11 November 2025)” and the RCSB Protein Databank “https://www.rcsb.org (accessed on 11 November 2025)”. The center positions of the grid box were determined based on the interaction between the compound and receptor Acsl4 (x = 2.86, y = −0.224, z = −0.076); GPX4 (X = 0.888, y = −0.736, z = −0.897); PDB ID:2dyh (x = 2.777, y = 1.498, z = 1.813); UniProt ID: Q9WTR6 (x = 1.873, y = 6.83, z = 10.866) and PDB ID: 1suv (x = 2.86, y = −0.224, z = −0.076). This grid box was defined to encompass the binding site of the target protein, where the small molecule interacts with key residues. Molecular docking was then performed in batches with 1.5.6 “http://mgltools.scripps.edu/down-loads/ (accessed on 11 November 2025)”. Protein and ligands in PDBQT format were prepared using Discovery Studio 2021 and AutoDock Tools 1.5.6 “http://mgltools.scripps.edu/down-loads/ (accessed on 11 November 2025)”. The top ligand-binding docking poses were ranked according to their binding energies/scores, and the predicted binding interactions were analysed using Discovery Studio 2020 Client.

### 2.3. Network Construction “Curcumin/Doxorubicin-Ferroptosis” Network

The canonical SMILES of CUR and DOX were obtained from the PubChem database and were used to predict potential targets using the Swiss Target Prediction, DrugBank, PharmMapper, and SuperPred. All predicted targets were combined, duplicates were removed, and protein identifiers were converted into official gene symbols using the UniProt database. Ferroptosis-related genes were obtained from FerrDb “https://www.zhounan.org/ferrdb/v3/pages/index.html (accessed on 20 December 2025)”, a curated ferroptosis-specific database comprising drivers, suppressors, and markers of ferroptosis. After removing duplicates, overlapping targets between (i) CUR and FerrDb ferroptosis gene; (ii) DOX and FerrDb ferroptosis gene; and (iii) CUR, DOX, and FerrDb ferroptosis genes were identified using an online Venn diagram tool. The overlapping genes were entered into the STRING database to construct protein–protein interaction (PPI) networks, using the following criteria: species: *Homo sapiens*, minimum interaction confidence score: ≥0.4 (medium confidence), and the number of protein interactions in the first shell: ≤10.Cytoscape 3.10.3 was used to visualize the PPI network of “DOX-ferroptosis targets”, “CUR-ferroptosis targets”, and “CUR/DOX-ferroptosis targets”. Whereby nodes represented the protein targets between drugs and ferroptosis, while edges showed the interactions between the protein targets. The topological analyses of the PPI network were measured using Cytoscape 3.10.3, with the focus on five parameters: (a) Degree, (b) Radiality, (c) Closeness centrality, (d) Stress, and (e) Betweenness. In this study, the 10 top nodes ranked by each parameter were selected and subsequently considered as hub targets for GO and KEGG pathway analyses. The hub targets were entered into the DAVID database for enrichment analyses of GO terms (biological process BP, cellular component CC, and molecular function MF) and KEGG pathways. Terms with *p* < 0.05 were considered statistically significant.

### 2.4. Cell Culture and Maintenance

The multiple-drug-resistant human lung epithelial cancer cell line, H69AR (CRL-11351), was purchased from the American Type Culture Collection (ATCC). H69AR cells were cultured in RPMI 1640 medium supplemented with 20% *v*/*v* FBS at 37 °C in a humidified incubator containing 5% CO_2_. Culture medium was changed every 2–3 days, and cells were passaged at 80–90% confluency.

#### 2.4.1. Cytotoxicity Assay

The cell viability following single or combination treatments with CUR and/or DOX was evaluated using MTS cytotoxicity. For single-agent treatments, 5 × 10^4^ cells/well were seeded onto 96-well plates overnight and treated with various concentrations of CUR or DOX (0–100 µM, final concentration) for 24 h. The IC_50_ values for each drug were calculated in GraphPad Prism 10.5 and used to select combinations for further testing. Combination treatments were evaluated by fixing CUR at sub-inhibitory concentrations (IC_10_, IC_20_, IC_30_) while varying the concentration of the DOX. Similarly, the combination of DOX of fixed sub-inhibitory concentrations (IC_10_, IC_20_, IC_30_) and varying concentrations of CUR was evaluated for MTS cytotoxicity. Subsequently, 20 µL of MTS reagent was added to each well and incubated for 4 h, after which absorbance was measured at 490 nm (Tecan Infinite M200 PRO, Männedorf, Switzerland).

#### 2.4.2. Reactive Oxygen Species (ROS) Assay

Intracellular ROS levels were quantified using 2′,7′-DCFA. Before treatments, 5 × 10^4^ cells/well were seeded onto 96-well plates and incubated overnight. Cells were then treated with either single agents or combination formulations for 24 h. To confirm the role of ROS in regulating cell death, cells were pretreated with 1 mM NAC for 24 h before CUR/DOX treatment. After washing, 100 µL of PBS containing 2′,7′-DCFA (10 µM) was added and incubated for 1 h at 37 °C in the dark. The supernatant was collected to measure the fluorescence intensity of dichlorofluorescein (Ex/Em: 485/538 nm) (Tecan Infinite M200 PRO, Switzerland).

#### 2.4.3. Adenosine Triphosphate (ATP) and Mitochondrial Membrane Potential (MMP) Assay

Quantifications of MMP and ATP levels in cells were done using the JC10 MMP assay kit (MAK159, Sigma-Aldrich, Malaysia) and the luminescent ATP detection assay kit (ab113849, Abcam, Cambridge, UK), respectively, as previously reported [10]. Briefly, for both ATP and MMP assays, 5 × 10^4^ cells/well were added onto 96-well plates and incubated overnight. Next, cells were treated with either single agents or combination formulations for 24 h. For MMP measurements, after treatment, cells were washed with pre-warmed PBS and stained with JC-10 dye based on the manufacturer’s protocols. The intensity of green fluorescence (Ex/Em: 490/525 nm) and red fluorescence (Ex/Em: 540/590 nm) was measured using Tecan Infinite M200 PRO, Männedorf, Switzerland. The intrinsic fluorescence of doxorubicin was corrected and subtracted from the corresponding samples. MMP depolarization was calculated based on the ratio between red and green fluorescence intensity. For ATP, treated cells were processed according to the protocols provided and the luminescence density was measured (FLUOstar^®^ Omega, BMG LABTECH, Ortenberg, Germany).

#### 2.4.4. Caspase-3 and Cytochrome C Assay

To measure caspase-3 and cytochrome C levels, cell lysates from H69AR cells treated with the formulations were prepared following published protocols [10]. Briefly, 1 × 10^6^ cells were added to the 6-well plates, incubated overnight and treated with the formulations for 24 h. After treatment, supernatant-free cells were collected via centrifugation and lysed using CelLytic^TM^ MT Cell lysis reagent (Sigma-Aldrich, Malaysia) with 1% (*v*/*v*) protease inhibitor. The samples were standardized to a protein concentration of 100 µg/mL. The concentrations of cytochrome C and caspase-3 were assayed according to the protocols provided with the ELISA kits (ab221832, Abcam, UK) and (ab39383, Abcam, UK), respectively [10]. For caspase-3, the fluorescence intensity was measured at Ex/Em: 400/505 nm (Tecan Infinite M200 PRO, Switzerland). For cytochrome C, the absorbance was measured at 550 nm using Tecan Infinite M200 PRO, Switzerland.

#### 2.4.5. Apoptosis, Cell Cycle, and DNA Fragmentation Assay

H69AR cells (5 × 10^5^) were seeded in a T25 flask and incubated overnight. Cells were pretreated with ferroptosis inhibitor FER-1 (1 µM) and/or ROS inhibitor NAC (1 mM) for 24 h, followed by exposure to CUR and/or DOX for another 24 h. The apoptosis measurement for the treated was conducted following the published protocols [10]. Cell cycle analysis of the treated cells was conducted based on previously published work [10]. The DNA fragmentation in cells following treatment with and without inhibitors was measured using the Cell Death Detection ELISA kit (11544675001, Roche, San Francisco, CA, USA) according to the manufacturer’s instructions.

#### 2.4.6. Lipid Peroxidation and Antioxidant-Related Enzymes Assay

Briefly, 1 × 10^6^ cells were added to the 6-well plates, incubated overnight and treated with the formulations for 24 h. Treated H69AR cells were lysed using CelLytic^TM^ MT Cell lysis reagent (Sigma-Aldrich, Malaysia) with 1% (*v*/*v*) protease inhibitor. The protein concentration in the cell lysate was determined with the BCA kit and standardized to 200 µg/mL with PBS. The proteins were measured using lipid peroxidation (ab118970), superoxide dismutase (ab309312, Abcam, UK), and glutathione (ab315322, Abcam, UK) according to the manufacturer’s protocol, and protein levels were normalised to the total protein content in the lysate [10]. Nuclear factor erythroid 2-related factor 2 (Nrf2, ab207223, Abcam, UK), glutathione peroxidase 4 (GPX4, ab304936, Abcam, UK), and thioredoxin reductase (TRx) contents were measured as per instructions from the manufacturers.

#### 2.4.7. Nuclear Factor Kappa B (NF-κB) and Phosphorylated Signal Transducer and Activator of Transcription-3 (p-STAT3) Assay, Multidrug Resistance-Associated Protein 1 (MRP1), and Iron (Fe^2+^) Concentration

Briefly, 1 × 10^6^ cells were added to the 6-well plates, incubated overnight and treated with the formulations for 24 h. The levels of NF-κB (ab279874, Abcam, UK), phosphorylated STAT-3 (p-STAT3, ab126459, Abcam, UK), and MRP1 (ELISA kit ab272211, Abcam, UK) were measured according to the manufacturer’s instructions. The absorbances of NF-κB, p-STAT3, and MRP1 were measured at 450 nm, 450 nm, and 450 nm, respectively. For the measurement of iron concentration, cells were pretreated with FER-1 (1 µM) for 24 h, followed by CUR and/or DOX treatment for 24 h. Cells were then lysed and further processed according to the iron assay kit instructions (ab83366, Abcam, UK).

### 2.5. Isobologram and Combination Index Analysis

The interaction between CUR and DOX was evaluated using the isobologram-based combination index (CI) method [20]. Briefly, H69AR cells were treated with CUR and DOX as single agents to determine their respective inhibitory concentrations (IC_10_, IC_20_, IC_30_, and IC_50_) based on MTS cell viability assays. For combination studies, one drug was fixed at sub-inhibitory concentrations (IC_10_, IC_20_, and IC_30_) while the concentration of the second drug was varied to achieve IC_50_. Two combination sets were employed: (a) fixed DOX concentration (IC_10_, IC_20_, and IC_30_) with varying CUR concentrations, and (b) fixed CUR concentration (IC_10_, IC_20_, and IC_30_) with varying DOX concentrations. CI < 1 indicates synergistic interaction; CI = 1 shows additive interaction; and CI > 1 shows antagonistic interaction. In addition, the drug combination data were analyzed using SynergyFinder version 3.0 “https://synergyfinder.fimm.fi (accessed on 26 October 2025)” [21]. Synergy was evaluated using four models: Bliss independence, Highest Single Agent (HSA), Loewe additivity, and Zero Interaction Potency (ZIP). Positive synergy scores indicate synergistic interactions, whereas negative scores indicate antagonism.

### 2.6. Statistical Analysis

Each experiment was performed in triplicate, and the data were expressed as mean ± standard deviation (SD). GraphPad Prism version 10.5 was used to perform a two-way analysis of variance (ANOVA). The statistical significance was determined using the Tukey test, and *p* < 0.05 was considered statistically significant.

## 3. Results

### 3.1. CUR and DOX Show Concentration-Dependent Synergistic Interaction in H69AR Cells

**Figure 1:**
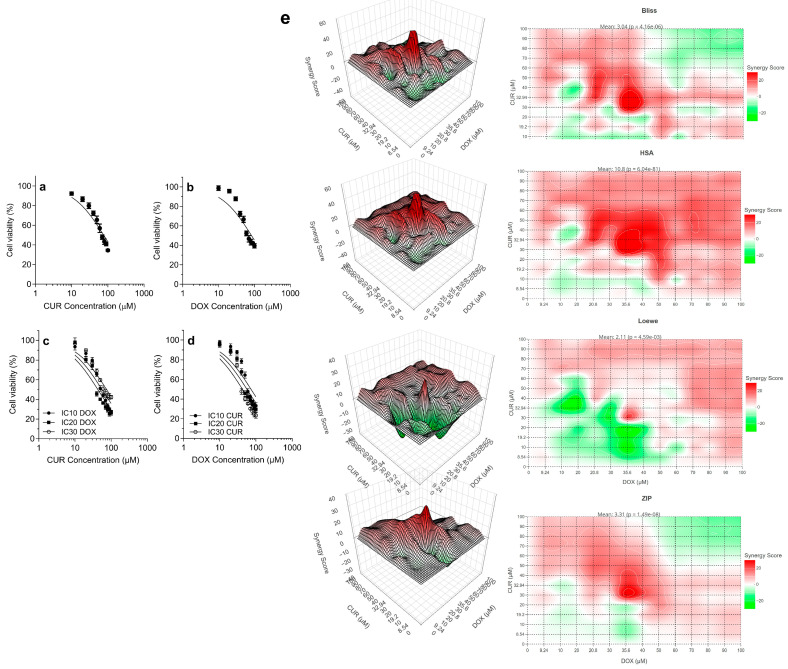
MTS assay of H69AR cells after 24 h treatment with (**a**) CUR alone and (**b**) DOX alone. (**c**) Cell viability following treatment with varying concentrations of CUR and a fixed concentration of DOX at IC_10_ (9.2 µM), IC_20_ (20.8 µM), and IC_30_ (35.6 µM). (**d**) Cell viability following treatment with varying concentrations of DOX and a fixed concentration of CUR at IC_10_ (8.5 µM), IC_20_ (19.2 µM), and IC_30_ (32.9 µM). (**e**) Three-dimensional (3D) synergy maps generated using SynergyFinder 3.0 by applying Bliss, HSA, Loewe, and ZIP models on CUR/DOX combination tested against H69AR cells. Data are expressed as mean ± SD (*n* = 3 independent experiments).

Figure 1a,b show the cytotoxic evaluation of CUR and DOX as single agents on the proliferation of H69AR cells. Single drug treatment with either CUR or DOX demonstrated a concentration-dependent reduction in cell viability. The IC_50_ results indicated that CUR (76.9 µM) was comparable with DOX (83.2 µM) against H69AR cells. Next, combination treatments were evaluated by fixing one agent at sub-inhibitory concentrations (IC_10_, IC_20_, IC_30_) while varying the concentration of the second agent (Figure 1c,d). When CUR dose–response curves were extrapolated in the presence of fixed DOX concentrations (IC_10_ = 9.2 µM, IC_20_ = 20.8 µM, and IC_30_ = 35.6 µM), the CUR concentration required to reach IC_50_ was 55.9 µM, 42.86 µM, and 36.64 µM, respectively (Figure 1c). In contrast, when H69AR cells were treated with fixed CUR concentrations at IC_10_ = 8.5 µM, IC_20_ = 19.2 µM, and IC_30_ = 32.94 µM, the DOX concentration required to achieve 50% cell death was 73.37 µM, 56.58 µM, and 42.7 µM, respectively (Figure 1d). The data indicated that when DOX (IC_10_–IC_30_) was combined with CUR, the amount of CUR required to reach IC_50_ was markedly lower than the concentration of DOX needed when combined with CUR (IC_10_–IC_30_). Based on the isobologram CI analysis (Table 1), the combination of DOX with CUR at fixed IC_10_, IC_20,_ and IC_30_ values, the CI values at IC_50_ were 0.993 (IC_10_ CUR), 0.930 (IC_20_ CUR), and 0.942 (IC_30_ CUR). For CUR combined with DOX at fixed IC_10_, IC_20,_ and IC_30_ values, the CI values at IC50 were 0.838, 0.808, and 0.905, respectively (Table 1), which indicated synergistic interactions according to standard CI criteria (CI < 1). For the combination therapy that yielded 10% cell inhibition (IC_10_), the CI values ranged from 1.727 to 4.370, indicating antagonistic interactions. Meanwhile, at IC_30_, the CI values ranged from 0.985 to 1.514, consistent with additive to antagonistic interactions. Drug interactions were next evaluated using SynergyFinder analysis across the CUR-DOX concentration matrix (Figure 1e). The mean synergy scores for the Bliss, HAS, Loewe, and ZIP models were 2.62, 9.32, 1.82, and 2.85, respectively. Based on Figure 1e, positive synergy scores (marked as red regions) were observed at intermediate to high dose CUR and DOX concentration combinations. However, low-dose regions showed low synergy scores, indicating additive or antagonistic interactions. Therefore, by integrating the MTS assays (Figure 1a–d), the isobologram-based CI analysis (Table 1), and modelling by SynergyFinder (Figure 1e), the interaction between CUR and DOX was concentration-dependent, with synergistic behavior observed at IC_50_-equivalent concentrations. It should be noted that at IC_10_ and IC_30_-equivalent combination concentrations (low % cell inhibitory levels), the CUR-DOX interactions were predominantly antagonistic. Therefore, subsequent in vitro assays were conducted using CUR-DOX combination regimens that achieved IC_50_-level growth inhibition. In these assays, DOX was fixed at IC_10_, IC_20_, or IC_30,_ and CUR was titrated to IC_50_, and vice versa, with CUR fixed at IC_10_, IC_20_, and IC_30_ with DOX titrated to IC_50_. For clarity, these combinations are referred to as IC_10_ DOX + CUR, IC_20_ DOX + CUR, and IC_30_ DOX + CUR, IC_10_ CUR + DOX, IC_20_ CUR + DOX, and IC_30_ CUR + DOX throughout the manuscript.

For combination studies, one drug was fixed at sub-inhibitory concentrations (IC_10_, IC_20_, and IC_30_) while the concentration of the second drug was varied to achieve IC_50_. Data are derived from three independent experiments (*n* = 3). CUR: curcumin; DOX: doxorubicin. CI refers to a combination index in which CI > 1 Antagonism; CI = 1 Additive effect; CI < 1 synergism.

### 3.2. CUR and DOX Combination Induces Mitochondrial Dysfunction and ATP Depletion

**Figure 2:**
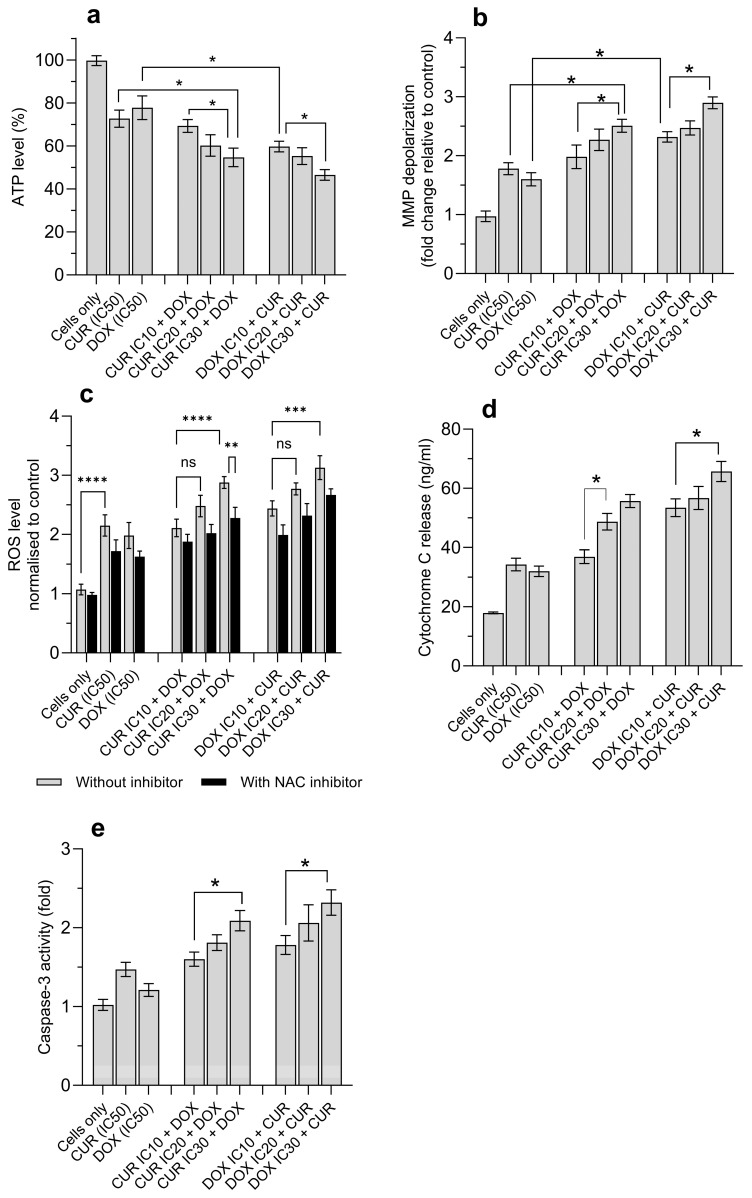
The combination treatment of DOX and CUR against H69AR cells after 24 h treatment. (**a**) ATP, (**b**) MMP depolarization, (**c**) ROS, (**d**) cytochrome C release, and (**e**) caspase-3 activity. Cells were treated with DOX and/or CUR at the following concentrations: DOX IC_10_ = 9.2 µM, IC_20_ = 20.8 µM, IC_30_ = 35.6 µM; CUR IC_10_ = 8.5 µM, IC_20_ = 19.2 µM, IC_30_ = 32.9 µM. Data are expressed as mean ± SD (*n* = 3 independent experiments). * Significantly different at *p* < 0.05 (Tukey post hoc test). ** Significantly different at *p* < 0.005 (Tukey post hoc test). *** Significantly different at 0.005 < *p* < 0.001 (Tukey post hoc test). **** Significantly different at *p* < 0.001 (Tukey post hoc test). n.s. non-statistically significant.

Given the synergistic effect on H69AR cell proliferation, we further examined the mechanistic effects of the combination treatment with DOX and CUR. As illustrated in Figure 2a, intracellular ATP levels in H69AR cells were significantly reduced following treatment with either drug alone or in combination. However, the reduction in ATP in H69AR cells treated with DOX and CUR alone was not statistically significant, with ATP values of 77.8 ± 5.5% and 72.7 ± 4.0%, respectively (Figure 2a). The combination treatment of CUR and DOX resulted in significantly higher concentration-dependent reduction in ATP levels. For example, reducing ATP levels was observed with the co-treatment of DOX with increasing CUR concentrations (IC_10_ to IC_30_) from 69.3 ± 3.0% to 54.7 ± 4.3% (Figure 2a). A similar but more significant effect was observed with increasing DOX concentrations (IC_10_ to IC_30_), where ATP levels declined from 59.7 ± 2.5% to 46.5 ± 2.5%.

Consistent with ATP depletion, MMP depolarization in cells increased following DOX and CUR treatment (Figure 2b). Approximately 1.6 ± 0.11- and 1.78 ± 0.1-fold MMP depolarization was noted in cells treated with DOX and CUR alone, respectively (Figure 2b). The combination treatment significantly increased MMP depolarization in a concentration-dependent manner. The MMP depolarization (fold vs. control) was 1.98 ± 0.20, 2.27 ± 0.18, and 2.51 ± 0.11 for IC_10_ CUR + DOX, IC_20_ CUR + DOX, and IC_30_ CUR + DOX, respectively. In contrast, for cells treated with combinations of CUR and DOX with varying IC values, significantly higher MMP depolarization in the cells was noted: IC_10_ DOX + CUR (2.32 ± 0.09), IC_20_ DOX + CUR (2.47 ± 0.12), and IC_30_ DOX + CUR (2.90 ± 0.1) (Figure 2b).

Higher ROS levels could contribute to higher cell death. Fluorescence intensity from the 2′,7′-DCFA reaction further confirmed that IC_30_ DOX + CUR and IC_30_ CUR + DOX resulted in significantly increased ROS production by more than 3.1-fold and 2.8-fold, respectively, and eventually contributed to higher cell death (Figure 2c). However, DOX alone produced a lower ROS level than CUR alone. We also pretreated the cells with NAC, a ROS scavenger, to further confirm the contribution of ROS in cell death. All treatment groups showed reduced intracellular ROS levels (Figure 2c).

To further assess whether MMP depolarization activated intrinsic apoptotic signaling, the concentration of cytochrome C (ng/mL) was measured (Figure 2d). Treatment with CUR alone or DOX alone resulted in a moderate increase in cytochrome C levels at 34.3 ± 2.1 ng/mL and 32 ± 1.8 ng/mL, respectively, compared to untreated cells (17.9 ± 0.3 ng/mL). The combination treatment significantly promoted cytochrome C release, particularly for the groups treated at IC30 values of CUR or DOX. For instance, IC_30_ CUR + DOX and IC_30_ DOX + CUR produced 55.7 ± 2.2 ng/mL and 65.7 ± 3.4 ng/mL, respectively (Figure 2d). This corresponded to 3.67- and 3.11-fold increases relative to control cells. This observation is consistent with the observed concentration-dependent activation of caspase-3 (Figure 2e). The caspase-3 (fold) activity followed the decreasing trend: IC_30_ DOX + CUR (2.32 ± 0.16) > IC_30_ CUR + DOX (2.09 ± 0.13) > IC_20_ DOX + CUR (2.06 ± 0.23) > IC_20_ CUR + DOX (1.81 ± 0.1) > IC_10_ DOX + CUR (1.78 ± 0.12) > IC_10_ CUR + DOX (1.6 ± 0.09). These findings indicate that caspase-3 activation increased with higher drug concentrations, irrespective of the type of drugs used.

### 3.3. CUR and DOX Induce Apoptosis with Involvement of ROS and Ferroptosis-Related Pathways

**Figure 3:**
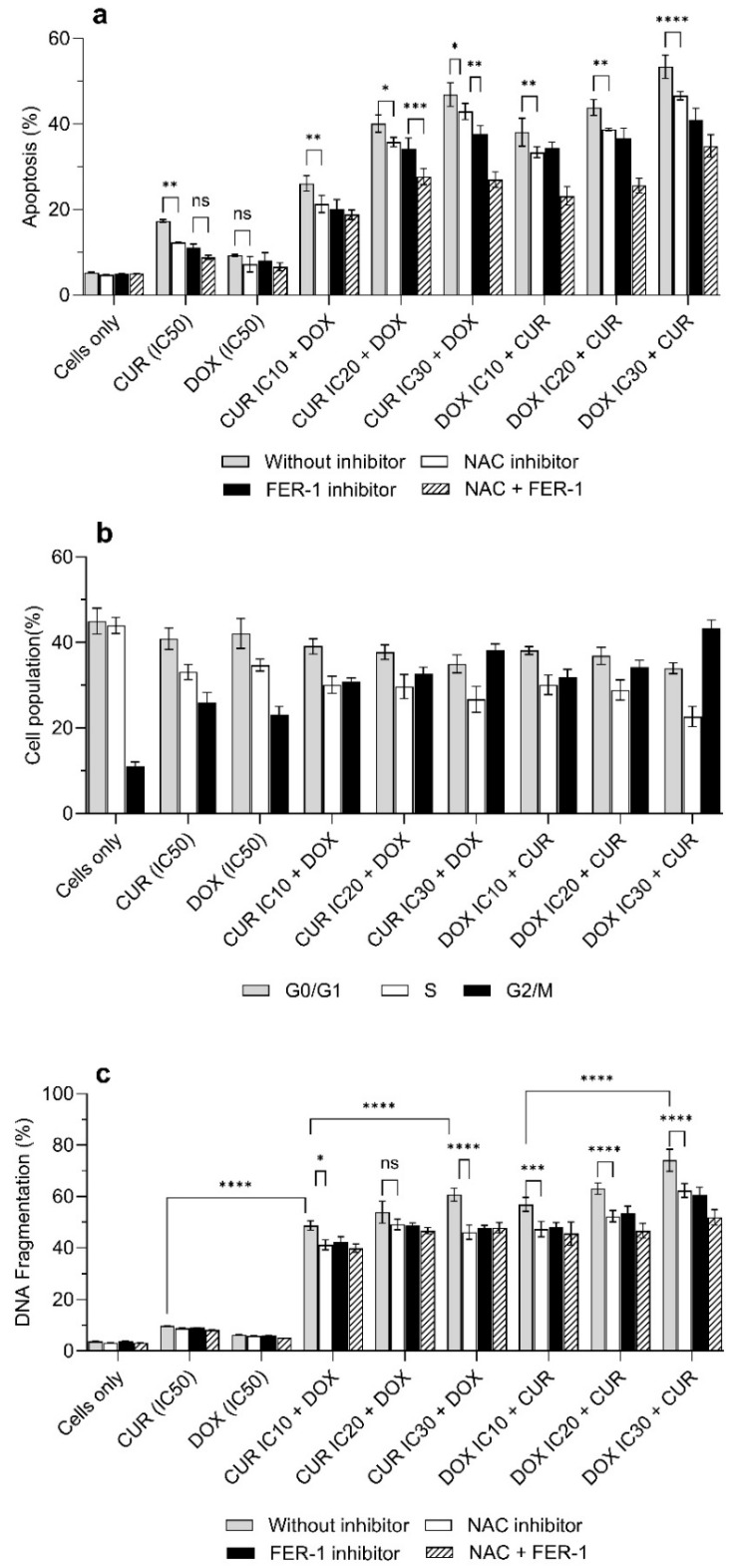
The combination treatment of DOX and CUR against H69AR cells after 24 h treatment. (**a**) Apoptosis, (**b**) Cell cycle analysis, and (**c**) DNA fragmentation. Cells were treated with DOX and/or CUR at the following concentrations: DOX IC_10_ = 9.2 µM, IC_20_ = 20.8 µM, IC_30_ = 35.6 µM; CUR IC_10_ = 8.5 µM, IC_20_ = 19.2 µM, IC_30_ = 32.9 µM. Data are expressed as mean ± SD (*n* = 3 independent experiments). * Significantly different at *p* < 0.05 (Tukey post hoc test). ** Significantly different at *p* < 0.005 (Tukey post hoc test). *** Significantly different at 0.005 < *p* < 0.001 (Tukey post hoc test). **** Significantly different at *p* < 0.001 (Tukey post hoc test). n.s. non-statistically significant.

The contribution of intracellular ROS in cell death has led us to explore its association with ferroptosis. For this purpose, we pretreated the cells with NAC, ferrostatin-1 (FER-1), or their combination before exposure to CUR and DOX. The DNA fragmentation in H69AR cells after treatment with either CUR alone or DOX alone was not significantly affected by pretreatment with inhibitors (Figure 3c). However, combination treatment clearly demonstrated the involvement of ROS and ferroptosis in regulating DNA fragmentation. More than 20% of the DNA fragmentation in H69AR cells was observed in the IC_30_ DOX + CUR treatment group after pretreatment with both ROS and ferroptosis inhibitors. No statistically significant differences were noted between treatment groups after pre-treatment with either NAC or FER-1 alone. However, all inhibitor pretreatments substantially reduced DNA fragmentation compared with untreated groups, thereby confirming the contribution of ROS- and ferroptosis-dependent mechanisms in cell death (Figure 3c). Apoptosis analysis further supports the role of ROS and ferroptosis in controlling proliferation in H69AR cells (Figure 3a). Inhibitor pretreatment significantly reduced the apoptotic cell populations across all combination treatment groups. Additionally, CUR alone was more effective than DOX alone to cause apoptosis in cells.

### 3.4. CUR and DOX Alter Ferroptosis-Related Oxidative Stress and Antioxidant Defense Systems

**Figure 4:**
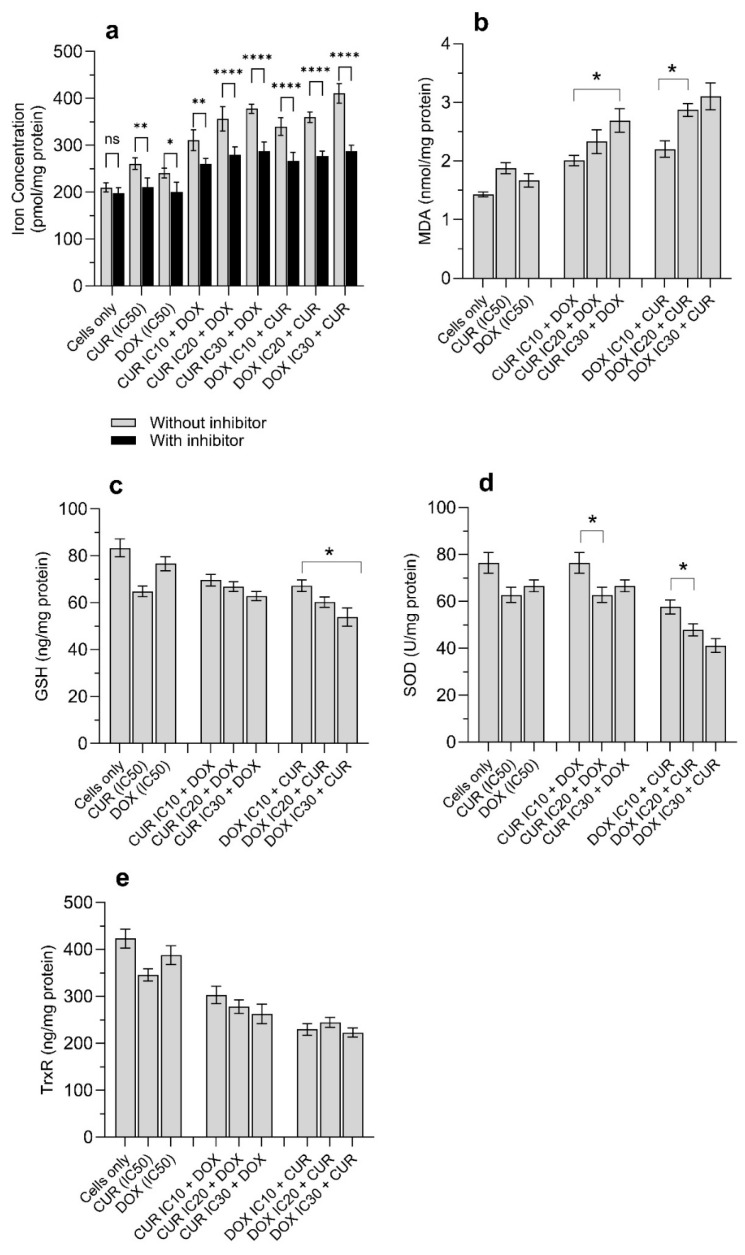
The combination treatment of DOX and CUR against H69AR cells after 24 h treatment. (**a**) iron concentrations, (**b**) MDA, (**c**) GSH, (**d**) SOD, (**e**) TrxR. Cells were treated with DOX and/or CUR at the following concentrations: DOX IC_10_ = 9.2 µM, IC_20_ = 20.8 µM, IC_30_ = 35.6 µM; CUR IC_10_ = 8.5 µM, IC_20_ = 19.2 µM, IC_30_ = 32.9 µM. Data are expressed as mean ± SD (*n* = 3 independent experiments). * Significantly different at *p* < 0.05 (Tukey post hoc test). ** Significantly different at *p* < 0.005 (Tukey post hoc test). **** Significantly different at *p* < 0.001 (Tukey post hoc test). n.s. non-statistically significant.

We further evaluated the intracellular iron levels and found that the iron levels in treated cells were generally higher than those in untreated cells (Figure 4a). Iron content increased from 210 ± 9.7 pmol/mg protein in untreated cells to 410 ± 20.9 pmol/mg protein following IC_30_ DOX + CUR treatment. Other combination treatments resulted in iron levels ranging from 310 ± 22.2 to 360 ± 11.3 pmol/mg of protein (Figure 4a). The pretreatment of cells with FER-1 reduced the overall intracellular iron concentration, and the effects were more pronounced with combination therapy compared to single drug treatment (Figure 4a). Cell death caused by dysregulation of iron concentration and lipid peroxidation is known as ferroptosis. Overall, the lipid peroxidation levels were markedly increased following the combination treatment of DOX and CUR compared to untreated cells (Figure 4b). The MDA level in H69AR cells treated with DOX alone and CUR alone ranged between 1.67 and 1.88 nmol/mg of protein. In contrast, the combination treatment of CUR concentration increasing from IC_10_ to IC_30_ resulted in significantly higher MDA levels, increased from 2.01 ± 0.09 to 2.69 ± 0.2 nmol/mg of protein. An increase of more than 40% in MDA levels was observed in cells when the concentration of DOX was increased from IC_10_ to IC_30_ (Figure 4b).

Next, we evaluated the concentrations of antioxidant enzymes in cells. Insufficient basal levels of antioxidant enzymes might result in elevated intracellular ROS levels, which eventually cause oxidative stress-associated cell damage. As shown in Figure 4c,d, the glutathione (GSH) and superoxide dismutase (SOD) levels decreased following treatment with either DOX alone or CUR alone. The intracellular levels of GSH and SOD were as follows: GSH (ng/mL)—untreated cells (83.40 ± 3.9) versus CUR treatment (64.90 ± 2.3) versus DOX (76.70 ± 3.0), and SOD (U/mg)—untreated cells (76.50 ± 4.4) versus CUR treatment (62.80 ± 3.3) versus DOX (66.60 ± 2.0). Combination treatment further reduced GSH and SOD levels in a concentration-dependent manner. When cells were challenged with IC_30_ DOX + CUR, the GSH and SOD levels were 53.9 ± 3.9 ng/mL and 41.8 ± 2.9 U/mg, respectively (Figure 4c,d). TrxR expression decreased with increasing treatment concentrations, although the changes were not statistically significant (Figure 4e).

### 3.5. CUR and DOX Modulate p-STAT3, and Reduce NF-κB, GPX4, Nrf2 and MRP1 Protein Levels

**Figure 5:**
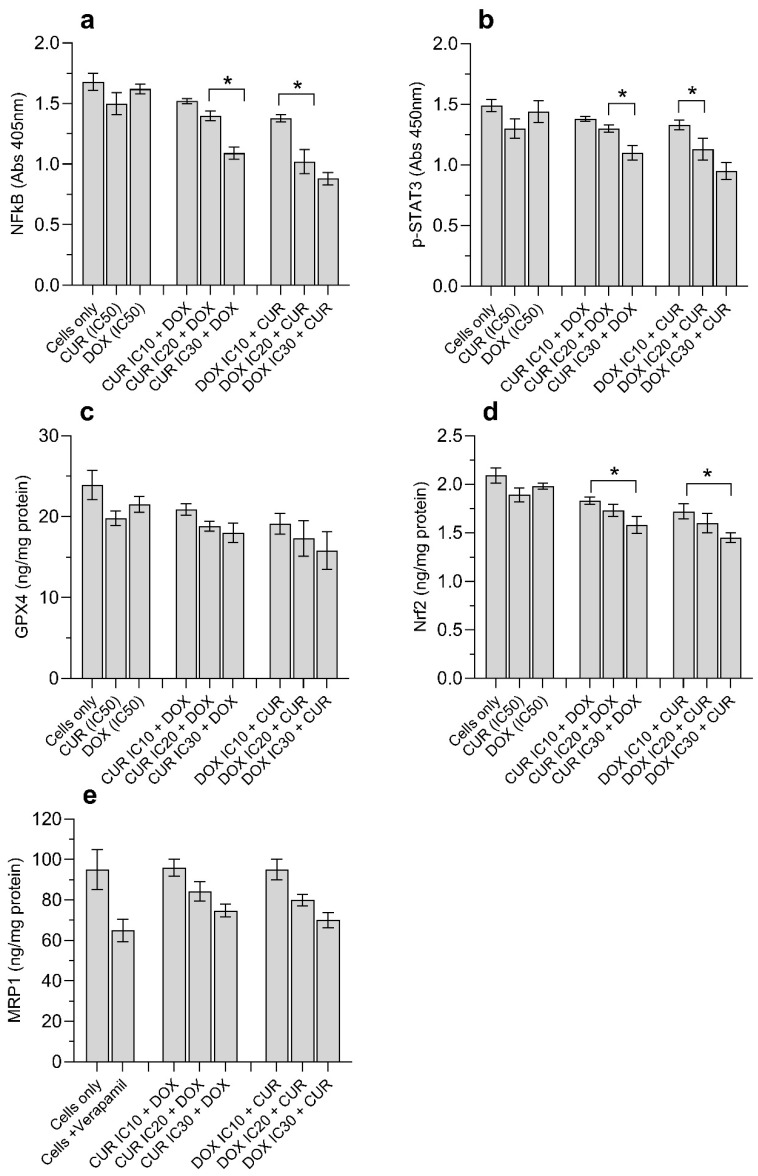
The combination treatment of DOX and CUR against H69AR cells after 24 h treatment. (**a**) NFKB, (**b**) p-STAT3, (**c**) GPX4, (**d**) Nrf2, and (**e**) MRP1. Cells were treated with DOX and/or CUR at the following concentrations: DOX IC_10_ = 9.2 µM, IC_20_ = 20.8 µM, IC_30_ = 35.6 µM; CUR IC_10_ = 8.5 µM, IC_20_ = 19.2 µM, IC_30_ = 32.9 µM. Data are expressed as mean ± SD (*n* = 3 independent experiments). * Significantly different at *p* < 0.05 (Tukey post hoc test).

To further understand the anti-proliferative mechanisms, we examined the expression of growth-related proteins (NF-κB and p-STAT3) in cells treated with DOX and CUR. We found that NF-κB (Figure 5a) protein levels and p-STAT3 (Figure 5b) in H69AR cells were reduced in a concentration-dependent manner. The most significant reductions were observed in cells treated with IC_30_ DOX + CUR, and IC_30_ CUR + DOX (Figure 5a,b). Alterations in SOD and GSH activities were associated with changes in GPX4 and Nrf2 expression. The combination treatment of IC_30_ DOX IC30 + CUR was more effective in inhibiting the expression of GPX4 levels compared to IC_30_ CUR + DOX (Figure 5c). The GPX4 protein levels (ng/mg of protein) decreased from 23.9 ± 1.8 (untreated cells) to 18.0 ± 1 (IC_30_ CUR + DOX) and 15.8 ± 2.3 (IC_30_ DOX + CUR). However, no significant differences were observed at lower concentrations (Figure 5c). Significant differences in Nrf2 levels were noted when cells were treated with increasing IC concentrations of either CUR or DOX (Figure 5d). Overall, the antioxidant system in H69AR cells responded negatively to CUR and DOX treatments, limiting the capacity to neutralize oxidative stress induced by these agents. We also showed that the combination of DOX and CUR was associated with reduced MRP-1 protein levels (Figure 5e). The MRP-1 levels were reduced from 95.0 ± 9.8 ng/mg protein (untreated cells) to 74.7 ± 3.2 ng/mg protein (IC_30_ CUR + DOX) and 70.1 ± 3.8 ng/mg protein (IC_30_ DOX + CUR).

### 3.6. Network Pharmacology and Molecular Docking Analyses Suggest Potential Ferroptosis-Related Associations for the CUR–DOX Combination

**Figure 6:**
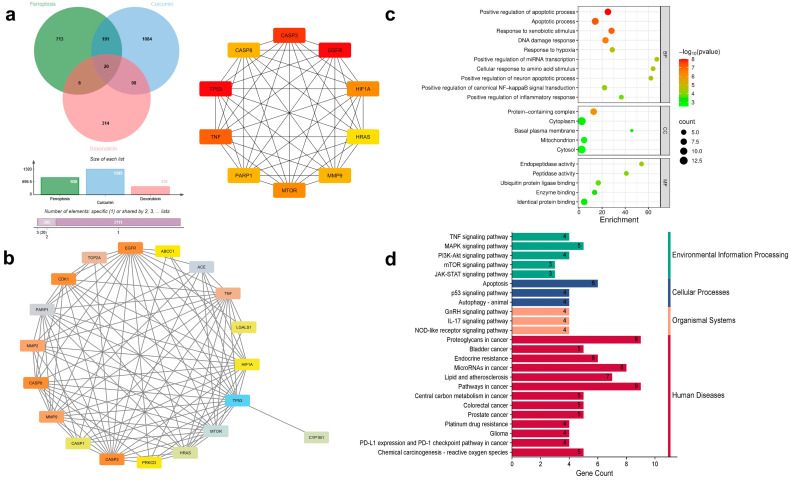
Network pharmacological prediction for lung cancer treated with CUR and DOX mapped to specific ferroptosis targets. (**a**) Venn diagram of ferroptosis genes target and CUR and DOX targets (**Left**). Example of the 10 top nodes identified for the “Degree” parameter using the Cytohubba plugin in Cytoscape (**Right**). (**b**) PPI networks for key therapeutic targets. (**c**) Analysis of potential targets of ferroptosis genes and CUR and DOX targets using gene ontology. (**d**) Analysis of KEGG enrichment pathways for drug-treated lung cancer. BP: biological process; CC: cellular component; MF: molecular function.

**Figure 7:**
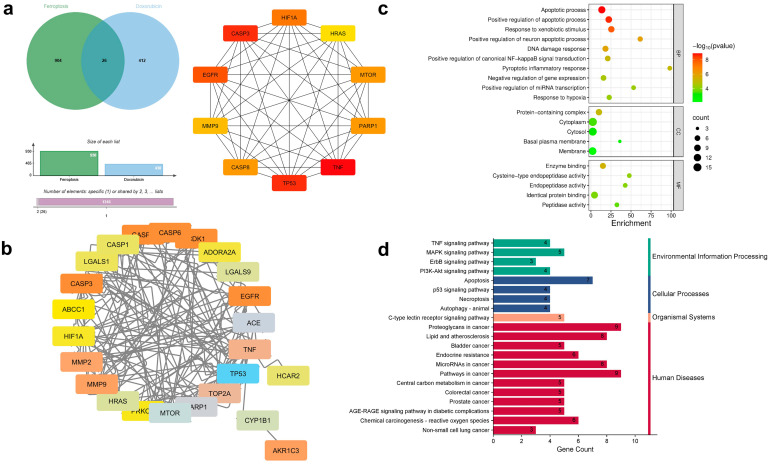
Network pharmacological prediction for lung cancer treated with DOX mapped to specific ferroptosis targets. (**a**) Venn diagram of the targeted ferroptosis genes and doxorubicin targets (**Left**). Example of the 10 top nodes identified for the “Degree” parameter using Cytohubba plugin in Cytoscape (**Right**). (**b**) PPI networks for key therapeutic targets. (**c**) Analysis of potential targets of ferroptosis genes and doxorubicin targets using gene ontology. (**d**) Analysis of KEGG enrichment pathways for drug-treated lung cancer. BP: biological process; CC: cellular component; MF: molecular function.

**Figure 8:**
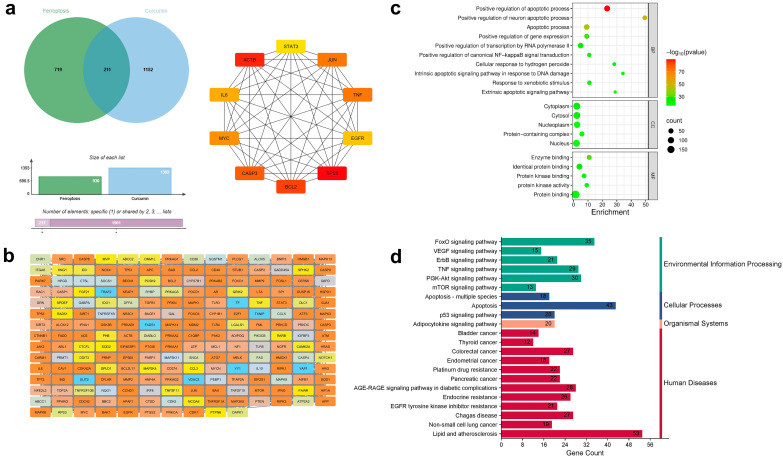
Network pharmacological prediction for lung cancer treated with CUR mapped to specific ferroptosis targets. (**a**) Venn diagram of the targeted ferroptosis genes and CUR targets (**Left**). Example of the 10 top nodes identified for the “Degree” parameter using the Cytohubba plugin in Cytoscape (**Right**). (**b**) PPI networks for key therapeutic targets. (**c**) Analysis of potential targets of ferroptosis genes and CUR targets using gene ontology. (**d**) Analysis of KEGG enrichment pathways for drug-treated lung cancer. BP: biological process; CC: cellular component; MF: molecular function.

To evaluate the association of ferroptosis genes in the regulation of cell death in resistant lung cancer cells, network pharmacology and molecular docking approaches were used. A total of 930 ferroptosis-related genes were retrieved from the FerrDb database. Drug-target predictions have identified 438 and 1393 associated targets for DOX and CUR. The overlapping genes analysis identified (a) 20 genes shared among ferroptosis-related genes, CUR, and DOX (Figure 6); (b) 26 genes between ferroptosis-related genes and DOX (Figure 7), and (c) 211 genes between ferroptosis-related genes and CUR (Figure 8). The 20 overlapping genes in the combination of CUR and DOX target include *HRAS*, *TP53*, *ABCC1*, *MTOR*, *HIF1A*, *MMP9*, *CASP1*, *CASP3*, *CASP8*, *CDK1*, *PARP1*, *TNF*, *ACE*, *MMP2*, *LGALS1*, *CYP1B1*, *PRKCD*, *TOP2A*, and *C1QBP* (Figure 6), indicating that CUR and DOX are involved in ferroptosis regulation. The Dox-only overlap comprised 26 targets, including *TP53*, *EGFR*, *MTOR*, *HIF1A*, *TNF*, *HRAS*, *PARP1*, *CASP3*, and *MMP9* (Figure 7). The overlapping genes were imported into the STRING database for protein–protein interaction (PPI) network. The CUR and DOX combination network contained 19 nodes and 101 edges, while the DOX network contained 24 nodes and 117 edges, and the CUR network comprised 206 nodes and 4795 edges. Using the CytoHubba plugin in Cytoscape, the top 10 hub targets were identified with degree centrality selected as the primary parameter to rank the nodes based on direct protein–protein interactions (Figure 6, Figure 7 and Figure 8). For the CUR and DOX-ferroptosis network, the top 10 hub genes were *TNF*, *HIF1A*, *HRAS*, *MMP9*, *MTOR*, *CASP8*, *EGFR*, *PARP1*, *TP53*, and *CASP3* (Figure 6) and their biological functions are summarized in Table 2. For the DOX-ferroptosis network, similar hub genes were identified, such as *TNF*, *CASP8*, *HIF1A*, *MMP9*, *MTOR*, *HRAS*, *PARP1*, *EGFR*, *TP53*, and *CASP3* (Figure 7). Meanwhile, the CUR-ferroptosis network demonstrated a broader protein-protein network with *IL6*, *EGFR*, *ACTB*, *STAT3*, *BCL2*, *JUN*, *CASP3*, *TP53*, *TNF*, and *MYC* identified as the top hub genes (Figure 8). The majority of the hub genes are associated with key regulators of oxidative stress, inflammatory signaling, apoptosis, and transcriptional regulation. Since the potential CUR or DOX therapeutic targets were almost identical, the potential mechanistic pathways could be similar. GO BP analysis revealed significant enrichment in apoptotic regulation, response to oxidative stress, DNA damage response, inflammatory signaling, hypoxia response, and regulation of ROS and metabolic processes (Figure 6, Figure 7 and Figure 8). Meanwhile, CC enrichment showed that the targets were predominantly localized at the cytoplasm, mitochondrion and nucleus. These compartments are closely associated with ROS generation, lipid peroxidation, redox signaling, and intracellular stress responses. The KEGG pathway analysis showed significant enrichment in pathways related to cancer, apoptosis, the MAPK signaling pathway, the p53 signaling pathway, the TNF signaling pathway, the HIF-1 signaling pathway, and chemical carcinogenesis-reactive oxygen species.

**Figure 9:**
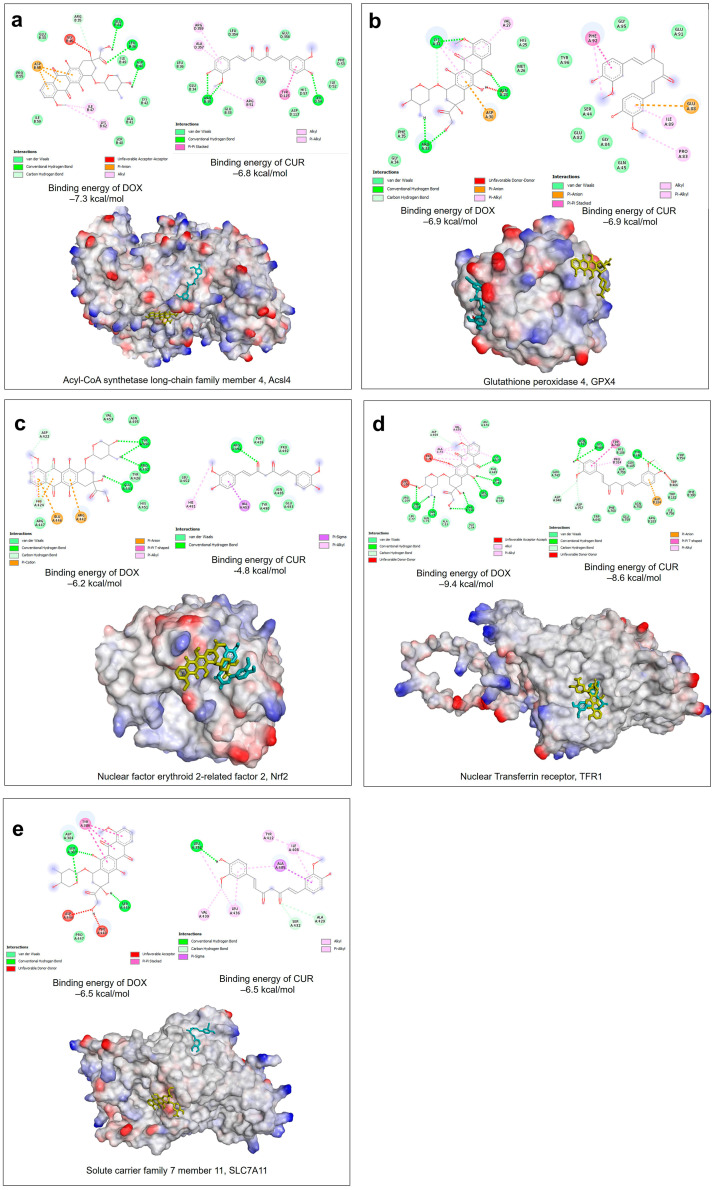
Orientation of DOX and CUR and their plausible binding interactions in the (**a**) Acyl-CoA synthetase long-chain family member 4, Acsl4 (PDB ID:8w0u), (**b**) Glutathione peroxidase 4, GPX4 (PDB ID 6hkq), (**c**) nuclear factor erythroid 2-related factor 2, Nrf2 (PDB ID:2dyh), (**d**) nuclear Transferrin receptor, TFR1 (PDB ID: 1suv), (**e**) Solute carrier family 7 member 11, SLC7A11 (UniProt ID: Q9WTR6) with adjacent residues binding site predicted from molecular docking. Note that the compounds in the residues are coloured as follows: DOX in yellow, CUR in blue.

DOX showed slightly higher binding affinity (−7.3 kcal/mol) compared to CUR (−6.8 kcal/mol) (Figure 9a) in Acsl4. The hydroxyl group of both compounds form hydrogen bond interactions with ASP46, GLU34, ARG35, and SER54. DOX stabilizes pi-anion interaction between the electron-deficient antraquinone ring and the negatively charged carboxylate side chain of ASP58, whereas the aromatic ring of CUR forms a more complex and extensive alkyl interaction with the hydrophobic side chains of ALA357, ARG359, TYR155 and ARG51. The GPX4 is a shallow catalytic pocket that resembles protein-protein interfaces more than classic deep druggable pockets. Selenocysteine at position 46 is the primary site for covalent inhibition. The docking results (Figure 9b) showed that CUR and DOX exhibited the same binding affinity of 6.9 kcal/mol but were positioned on the opposite sides within the GPX4 binding pocket. The benzene ring of DOX and CUR forms a pi-anion with ASP30 and Glu88, respectively. DOX forms a hydrogen interaction with LYS31, ASN28, and ARG33. CUR primarily forms van der Waals forces with the amino acid residues surrounding active binding sites, suggesting that both compounds have a potential advantage for achieving selectivity, which would inhibit GPX4’s function. DOX and CUR are both located in the same binding region of the Nrf2 protein (Figure 9c), where DOX has a higher affinity (−6.2 kcal/mol) compared to CUR (−4.8 kcal/mol). This could be mainly due to the stronger pi-anion (GLU446 and ARG442) and hydrogen interaction (GLU444, PRO492, and TYR490) forming between doxorubin and amino acid residues in the binding pocket. For CUR, only the carbonyl group from the center linker forms a hydrogen bond with ARG442, which is further stabilized by the relatively weak hydrophobic interactions. The docking results (Figure 9d) between DOX and CUR with TRF1 showed that both compounds targeted the same binding region, with DOX (−9.4 kcal/mol) having slightly higher affinity compared to CUR (−8.6 kcal/mol). Hydrogen intermolecular forces become the primary binding interactions between DOX and the hydroxyl group of SER255, ASN76, PHE396, ARG668, VAL397, and LYS673 amino acid residues. There are no pi-anion interaction forms between the ring structure of DOX and TRF1. The results of the present molecular docking analysis (Figure 9e) indicated that CUR and DOX exhibited moderate binding capacity with SLC7A11, with binding energies of −6.50 kcal/mol for both compounds. Both compounds form hydrogen bond interactions with the amino acids SER387, SER494 and LEU398 at different binding regions of the SLC7A11 protein. Furthermore, both compounds were found to form favourable hydrophobic interactions with TYR386, TYR412, ILE408, VAL439 and LEU436, making an important contribution to the stabilization of small molecules.

## 4. Discussion

The side effects of chemotherapy in cancer treatment are well-documented and are unavoidable. Despite advances in targeted therapies and immunotherapies, chemotherapeutic agents remain the standard therapeutic modality for lung cancer in both early and late-stage diseases [22]. However, long-term repetitive use of chemotherapeutic agents is hampered by dose-limiting toxicity and the development of multidrug resistance (MDR). The mechanisms of MDR include increased drug efflux, immunoediting, sustained proliferative signaling, alterations in drug metabolism, changes in the tumor microenvironment, and metabolic reprogramming [23]. These survival mechanisms synergistically allow cancer cells to adapt dynamically to treatments. Given the tendency of cancer cells to develop drug resistance to chemotherapeutic drugs, the discovery of novel drugs or improvements to current treatment modalities is imperative to improve overall survival in lung cancer.

Plant-based compounds are gaining attention owing to their pleiotropic biological activities and ability to modulate multiple signaling pathways simultaneously. Majorities of plant-derived compounds possess both anti-inflammatory and anti/pro-oxidant properties, which are critical to modulate inflammation and oxidative stress-related pathways [24]. CUR, a well-known dietary polyphenol, can effectively sensitize and overcome chemoresistance in various cancer cell lines, such as DOX-resistant breast cancer cells [25], 5-fluorouracil-resistant colon cancer cells [26], daunorubicin-resistant acute myeloid leukaemia cells [27], and docetaxel-resistant prostate cancer cells [28]. These findings are due to its ability to target multidrug resistance proteins, such as P-gp, MRP1, and lung cancer resistance protein (LRP), which enhance the retention of chemotherapeutic agents within cells and ultimately increase cell death [10,29,30]. We have previously demonstrated that CUR inhibited the expression of MDR proteins, which increased the intracellular retention of CUR and ultimately led to cell death [10].

The ability of CUR to sensitize cancer cells to chemotherapeutic drugs means that it can be used in combination with conventional medicines to treat resistant lung cancer cells. In this study, it was demonstrated that CUR acted as a functional chemosensitizer, enhancing DOX toxicity to DOX-resistant lung cancer cells through concentration-dependent synergistic interactions (Figure 1, Table 1). Our results showed that the combination of CUR and DOX displayed antagonistic or additive interactions at low inhibitory levels (IC_10_ and IC_30_) with a clear transition toward additive-to-synergistic behavior at IC_50_-equivalent concentrations. When CUR and DOX were used at concentrations that caused approximately 10% growth inhibition (IC_10_), the combinations consistently showed antagonistic effects, as reflected by CI values above 1 (Table 1). It is possible that the mild stress or toxic effects induced by low-dose CUR-DOX combinations were easily compensated by the active, adaptive survival mechanisms of H69AR cells, which include antioxidant capacity, effective DNA repair, and active drug efflux. In other words, sublethal stress induced by low-dose combinations was insufficient to kill cancer cells. However, when IC_50_-equivalent concentrations of the CUR-DOX combinations were used, isobologram analysis (Table 1) and Synergy models (Bliss, HSA, Loewe, and ZIP) consistently supported synergistic interactions (Figure 1e). This indicated that the high-dose combinations resulted in the failure of compensatory mechanisms in H69AR cells and activation of irreversible cell death pathways. In addition, the synergism behaviour also showed that CUR did not enhance the DOX toxicity in an additive manner but instead acted to lower the cellular stress tolerance threshold required to activate death mechanisms. When DOX was fixed at sub-inhibitory concentrations (IC_10_, IC_20_, and IC_30_) and CUR was titrated, the CUR concentration required to reach IC_50_ was significantly lower than the concentration of DOX needed in a similar treatment design. Our data showed that CUR was more effective than DOX in reducing the concentration required to achieve IC_50_, suggesting that CUR acted to sensitize DOX-resistant cells rather than merely functioning as a co-cytotoxic agent. In our previous work, CUR was effective in killing H69AR cells through oxidative-stress-induced mitochondrial damage, which may contribute to the synergistic effects observed in this study.

CUR also significantly upregulated the expression of pro-apoptotic proteins (p53, Bax) and simultaneously suppressed the expression of anti-apoptotic proteins (Bcl-2 and Bcl-xL) [31]. The shift in apoptotic protein activation promotes mitochondrial destabilization, cytochrome C release, activation of caspases 3 and 9 to execute apoptotic cell death. In addition, CUR inhibited the activity of transcriptional regulators (NF-κB and COX-2) that are involved in inflammation-associated survival signaling and resistance [32]. However, apoptosis alone is often insufficient to eliminate MDR cancer cells, as resistance is commonly associated with suppression of apoptotic signaling pathways. Therefore, it is possible that CUR and DOX were involved in the activation of other regulated cell death mechanisms. Ferroptosis, an iron-dependent oxidative cell death, is driven by excessive iron accumulation beyond redox homeostatic thresholds, leading to uncontrolled accumulation of lethal lipid peroxidation and ROS [18]. Under normal conditions, cellular redox balance is maintained by a tightly regulated antioxidant system comprising GSH, GPX4, SOD, and TrxR. Naturally, higher levels of GSH and SOD are frequently observed in cancer cells [33]. When cancer cells are exposed to chemotherapeutic stress, it leads to the activation of GSH to maintain mitochondrial and nuclear membrane integrity, thereby supporting the continued proliferation of cancer cells. On the other hand, SOD reduced ROS-induced DNA damage, enabling tumor cells to adapt to oxidative stress [33]. Our previous work showed that CUR nanoparticles disrupted redox homeostasis in DOX-resistant H69AR lung cancer cells, as evident by significant depletions of GSH and SOD [10]. We also found that the collapse in the antioxidant buffering capacity was accompanied by increased lipid peroxidation, lysosomal oxidation, DNA instability, and protein oxidation [10]. These findings suggest that CUR directly disrupted the redox buffering systems that sustain MDR survival. This finding is in accord with our data, whereby the antioxidant enzymes (SOD and GSH) were significantly decreased following DOX or CUR treatment, and this effect was more pronounced when DOX and CUR were applied simultaneously to H69AR cells. This further supports the earlier observation whereby at IC_50_-equivalent concentrations, the combined effects of CUR-mediated antioxidant depletion and DOX-induced oxidative damage accumulate non-linearly and eventually cause the collapse of redox buffering capacity. When antioxidant defences are overwhelmed, excessive ROS accumulation and iron-dependent lipid peroxidation might trigger higher ferroptosis cell death (Figure 2 and Figure 3). In addition, at low-dose inhibitory concentrations, the combination treatments were antagonistic, suggesting that the low levels of oxidative and metabolic stress induced under such conditions were still within the oxidative neutralization/buffering capacity of H69AR cells. In other words, such sub-inhibitory conditions further activated the redox-dependent survival mechanisms, such as enhanced antioxidant enzyme activity and efficient lipid peroxide detoxification, in H69AR cells, in which the levels were sufficient to maintain the redox balance. This finding was confirmed using our combination of curcumin nanoparticles, reducing the cytotoxicity effect of paclitaxel nanoparticles towards Beas-2B by improving the antioxidant capacity [34]. The transition from antagonistic to synergistic effects with increasing IC concentrations supports a redox stress threshold-dependent interaction, whereby once the threshold is reached, the available antioxidant enzymes are unable to repair cellular stress, leading to cell death.

Previous studies have demonstrated that CUR upregulated the expression of heme oxygenase-1 (HO-1), leading to enhanced heme degradation and increased iron levels, and eventual ferroptotic cell death [35,36]. In addition, Li et al. found that the increase in intracellular iron levels was accompanied by enhanced ROS accumulation, increased lipid peroxidation, and impairment of glutathione activity [35]. Tang et al. showed that in vitro and in vivo CUR treatment caused lipid damage, iron accumulation, and ferroptosis in non-small cell lung cancer models [18]. Interestingly, Rainey et al. showed that CUR possesses iron-chelating properties, in which the complexation with metal ions through its β-diketone structure resulted in the loss of cytotoxic activity of CUR [37]. However, in this present study, both isobologram analysis (Table 1) and multiple synergy models (Bliss, HSA, Loewe, and ZIP; Figure 1e) demonstrated that synergistic interactions for CUR and DOX combination occurred at IC_50_-equivalent concentrations. This suggests that the enhanced cytotoxicity observed at these doses was not attributable to CUR alone, but rather to cooperative interactions between CUR and DOX. The establishment of a synergistic threshold at IC_50_-equivalent concentrations suggests that DOX contributes to overcoming cellular stress-compensatory mechanisms, thereby maintaining the cytotoxic activity of CUR despite its known iron-chelation behavior.

Concurrent inhibition of NF-κB and STAT3 further disrupted stress-adaptive and pro-survival signaling pathways. Downregulation of MRP1 additionally reduced efflux-mediated resistance, reinforcing intracellular drug accumulation and oxidative stress. Activation of NF-κB increased the production of anti-apoptotic and MDR proteins in cancer cells, eventually leading to sustained proliferation, metastasis, and drug resistance in cells [38]. In a previous study, CUR and its derivatives suppressed the activation of HER2 and NF-κB and chemosensitized the DOX-resistant breast cancer cells towards DOX [39]. We have recently demonstrated that CUR nanoparticles inhibited the NF-κB expression in H69AR cells in a size- and concentration-dependent manner [10]. Many studies have shown that inhibition of NF-κB is beneficial in blocking the activation of tumor-promoting genes such as TNFα, Bcl-2, Bcl-xl, cyclin D1, p53, COX-2, and IL-6 [40,41]. Similar to previous studies, we found that the combination treatment of CUR and DOX effectively reduced the cellular protein levels of NF-κB and MRP-1, accompanied by higher levels of apoptotic and ferroptotic cell death. We hypothesize that the downregulation of MRP-1 proteins blocked the drug efflux and increased intracellular accumulation of DOX and CUR. In turn, the high levels of DOX and CUR within cancer cells caused the release of calcium ions from the endoplasmic reticulum, which entered mitochondria to disrupt the electron transport chain and resulted in excessive ROS generation as well as ATP and MMP depolarization. This, in turn, activated the apoptotic-associated signaling pathways leading to massive cell death. In addition, the oxidative stress and lipid peroxidation induced within the cells were not adequately counteracted by cancer cell antioxidant systems, thereby triggering ferroptotic cell death. This is supported by a recent finding whereby CUR was mainly localized at the endoplasmic reticulum and associated with organelle swelling, combinations of an unfolded protein response and calcium release [42]. In addition, Zeng et al. demonstrated that DOX is localized in the endoplasmic reticulum and mitochondria in MCF-7/ADR cells [43].

Network pharmacology analysis provided mechanistic support that the CUR and DOX combination could enhance the anti-cancer effect by targeting coordinated modulation of multiple molecular pathways through ferroptosis. Although ferroptosis regulators such as GPX4 and SLC7A11 were not identified as top-degree hubs, many of the hub genes identified, such as TP53, TNF, HIF1A, MTOR, CASP3, CASP8, and PARP1, are functionally linked to the regulation of ferroptosis. These genes are responsible for regulating redox signaling, oxidative stress regulation, metabolic stress adaptation, mitochondrial dysfunction, hypoxia signaling, and DNA damage and cell death, which are inherently linked to ferroptosis activation. We have also mapped the overlapping targets of CUR-Ferroptosis, DOX-Ferroptosis, and CUR-DOX-ferroptosis to FerrDb classifications. Although not identified as top-degree hubs, both CUR and DOX were found to regulate ferroptosis drivers (e.g., ACSL4, TP53) and suppressors (e.g., GPX4, SLC7A11). In addition, central hub genes such as TP53, TNF, and PARP1, identified as ferroptosis drivers or positive regulators in DOX-ferroptosis networks, further support the contribution of oxidative stress and inflammation cell death mechanisms. On the other hand, HIF1A, MTOR, EGFR, STAT3, and BCL2 are identified as ferroptosis suppressors or modulators in FerrDb, suggesting the involvement of adaptive and compensatory antioxidant stress-response. Docking analyses showed that CUR and DOX displayed favourable binding affinities toward key ferroptosis regulators, including ACSL4 (lipid peroxidation driver), GPX4 (lipid peroxide detoxifier), NRF2 (antioxidant transcriptional regulator), TFR1 (iron uptake), and SLC7A11 (cystine transport). Importantly, CUR and DOX exhibited complementary binding profiles, suggesting cooperative disruption of multiple ferroptosis checkpoints.

While the present study demonstrates useful mechanistic insights, several limitations should be noted. First, the mechanistic data are based primarily on functional assays and ELISA-based quantification of total proteins rather than Western blot or qPCR analyses. While these assays provide quantitative screenings of treatment-induced changes, they do not directly measure transcriptional activation and post-translational states. Secondly, the study was conducted in a single DOX-resistant H69AR cell lines and therefore the present data should be interpreted as sensitization of a resistant cancer cell model using CUR. Third, given the limited solubility and bioavailability of CUR, in vivo validation will be essential to confirm the involvement of ferroptosis in the future. Despite the limitations, the study provides mechanistic insights linking ferroptosis, oxidative stress and mitochondrial dysfunction to chemosensitize drug-resistant lung cancer cells to treatment.

## 5. Conclusions

This study aimed to enhance the response of DOX against DOX-resistant lung cancer cells by sensitizing the cells with CUR. The results showed that CUR was effective in enhancing the DOX toxicity in a concentration-dependent manner through oxidative-stress and ferroptosis-apoptosis-associated cell death. A low-dose combination of CUR and DOX resulted in antagonistic interaction, probably attributable to the efficient antioxidant capacity within H69AR cells neutralizing the redox imbalance at the observed concentrations. Consequently, H69AR cells survived the low ROS insult. On the other hand, at higher dose combinations, which are IC_50_-equivalent concentrations, the oxidative stress and lipid peroxidation exerted on H69AR cells exceeded the toxic threshold, leading to irreversible mitochondrial damage, activation of apoptotic and ferroptotic-signaling pathways and eventually cell death. The combination treatment suppressed key proteins involved in cell proliferation, such as NF-κB and STAT3, thereby increasing overall apoptosis. The level of MRP-1 expression was also affected by the combination treatment, which may be linked to the induction of ferroptosis. Further research is necessary to explore the relationship between NF-κB, STAT3, MRP-1, ferroptosis, and apoptosis. Nonetheless, this study provides insights into the potential use of polyphenols, such as curcumin, to overcome multidrug resistance in cancer cells by modulating oxidative stress and ferroptosis targeting.

## Figures and Tables

**Table 1:** Isobologram-based combination index (CI) analysis of the interaction between CUR and DOX in H69AR cells based on MTS assays.

Sample	IC_10_ (µM)		CI	IC_30_ (µM)		CI	IC_50_ (µM)		CI
	CUR	DOX		CUR	DOX		CUR	DOX	
Single drug									
CUR alone	8.5	-	-	32.9	-	-	76.9	-	-
DOX alone	-	9.2	-	-	35.6	-	-	83.2	-
Combination drug									
DOX + CUR (IC_10_)	8.5	8.2	1.882	8.5	31.4	1.142	8.5	73.4	0.993
DOX + CUR (IC_20_)	19.2	6.3	2.928	19.2	24.2	1.263	19.2	56.6	0.930
DOX + CUR (IC_30_)	32.9	4.7	4.370	32.9	18.3	1.514	32.9	42.7	0.942
CUR + DOX (IC_10_)	6.2	9.2	1.727	23.9	9.2	0.982	55.9	9.2	0.838
CUR + DOX (IC_20_)	4.8	20.8	2.808	18.4	20.8	1.142	42.9	20.8	0.808
CUR + DOX (IC_30_)	4.1	35.6	4.329	15.7	35.6	1.477	36.6	35.6	0.905

**Table 2:** Top 10 hub genes identified from CUR-DOX-ferroptosis network and their relevance to ferroptosis based on FerrDb annotations.

Gene	Full Name	Relevance to Ferroptosis (FerrDb)
*TNF*	Tumor necrosis factor	Functionally linked to ferroptosis-associated and regulated cell death pathways
*HIF1A*	Hypoxia-inducible factor 1 alpha	Validated regulator of ferroptosis sensitivity via lipid metabolic pathways
*HRAS*	Harvey rat sarcoma viral oncogene homolog	Context-dependent ferroptosis-associated regulator
*MMP9*	Matrix metalloproteinase 9	Validated ferroptosis-associated regulator
*MTOR*	Mechanistic target of rapamycin	Validated suppressor of ferroptosis
*CASP8*	Caspase-8	Multi-pathway regulated cell death hub
*EGFR*	Epidermal growth factor receptor	Context-dependent ferroptosis regulator
*PARP1*	Poly(ADP-ribose) polymerase 1	Validated driver and regulator of multiple regulated cell death pathways
*TP53*	Tumor protein p53	Validated driver and regulator of multiple regulated cell death pathways
*CASP3*	Caspase-3	Central effector of apoptosis and multi-pathway regulated cell death

## Data Availability

The original contributions presented in this study are included in the article. Further inquiries can be directed to the corresponding authors.

## Abbreviations

ACSL4	Acyl-CoA synthetase long-chain family member 4
ATP	Adenosine triphosphate
BCA	Bicinchoninic acid
CI	Combination index
CUR	Curcumin
DCFH-DA	2′,7′-Dichlorodihydrofluorescein diacetate
DOX	Doxorubicin
ELISA	Enzyme-linked immunosorbent assay
ER	Endoplasmic reticulum
FER-1	Ferrostatin-1
GSH	Glutathione
GPX4	Glutathione peroxidase 4
H69AR	Doxorubicin-resistant human small cell lung cancer cell line
HIF1A	Hypoxia-inducible factor 1 alpha
HSA	Highest Single Agent model
KEGG	Kyoto Encyclopedia of Genes and Genomes
MDA	Malondialdehyde
MDR	Multidrug resistance
MMP	Mitochondrial membrane potential
MRP1 (ABCC1)	Multidrug resistance-associated protein 1
MTOR	Mechanistic target of rapamycin
NAC	N-acetyl-L-cysteine
NF-κB	Nuclear factor kappa B
NRF2 (NFE2L2)	Nuclear factor erythroid 2–related factor 2
PBS	Phosphate-buffered saline
PPI	Protein–protein interaction
p-STAT3	Phosphorylated signal transducer and activator of transcription 3
ROS	Reactive oxygen species
SLC7A11 (xCT)	Solute carrier family 7 member 11
SOD	Superoxide dismutase
STRING	Search Tool for the Retrieval of Interacting Genes/Proteins
TFR1	Transferrin receptor 1
TrxR	Thioredoxin reductase
ZIP	Zero Interaction Potency model
